# Normalization Strategies for miRNA RT-qPCR in Blood-Derived Samples in Obesity Research: Current Practices and Methodological Considerations

**DOI:** 10.3390/ijms27209079

**Published:** 2026-10-13

**Authors:** Anna V. Pustovalova, Alexandra F. Nikolaeva, Alexey I. Kalinkin, Vladimir O. Sigin

**Affiliations:** Research Centre for Medical Genetics, 115522 Moscow, Russia; a_pustovalova02@inbox.ru (A.V.P.); nikolaevaepi@gmail.com (A.F.N.); alexeika2@yandex.ru (A.I.K.)

**Keywords:** microRNA, reference genes, obesity

## Abstract

Reverse transcription quantitative PCR (RT-qPCR) is widely used to quantify circulating microRNAs, but reliable interpretation depends on appropriate data normalization. In obesity research, selecting reference RNAs for measurements in whole blood, blood cells, plasma, and serum is challenging because of biological heterogeneity and preanalytical variability. This review summarizes normalization strategies used in RT-qPCR studies of blood-derived samples from participants with overweight or obesity. We examine commonly used endogenous reference RNAs, including U6 snRNA and miR-16-5p, and assess the rationale for their selection and the available validation evidence in relation to sample matrix and cohort characteristics. The review evaluates normalization practices and the quality and reporting of reference-RNA validation in obesity-related studies, with particular attention to the biological comparison being made. No candidate, algorithmic ranking, or fixed panel size is considered universally applicable. Candidate selection, the number of references, and the normalization scale should be justified for the intended comparison. The review provides practical guidance for designing and reporting validation of reference small non-coding RNAs and process controls in study cohorts with overweight or obesity, with recommendations qualified by the available evidence.

## 1. Introduction

Obesity is a major public health concern. According to the World Health Organization, an estimated 35 million children under five years of age were overweight in 2024 [1]. Obesity is a multifactorial chronic disease characterized by excessive adipose tissue accumulation and associated with an increased risk of type 2 diabetes mellitus, cardiovascular disease, non-alcoholic fatty liver disease, and certain cancers [2,3]. Its pathogenesis involves genetic, epigenetic, behavioral, and environmental factors, including disturbances in energy balance, adipose tissue inflammation, and insulin resistance [3]. Regulation of genes involved in energy homeostasis is therefore an important area of investigation. MicroRNAs (miRNAs) are small non-coding RNAs, approximately 22 nucleotides long, that regulate gene expression at the post-transcriptional level [4]. They influence a broad range of cellular processes, including differentiation, proliferation, apoptosis, and development [5,6]. In obesity, miRNAs participate in adipocyte differentiation, lipid metabolism, and inflammatory responses [7]. Dysregulated miRNA expression may consequently contribute to obesity and its associated metabolic disorders [8].

MiRNAs in blood-derived samples are of particular interest as potential biomarkers. Whole blood and blood-cell fractions contain substantial cellular miRNA populations, whereas plasma and serum measurements include extracellular miRNAs. Their potential roles in obesity and related disorders have stimulated studies of bariatric surgery, adipogenesis, and metabolic regulation [5,9,10,11,12]. Available measurement platforms include small RNA sequencing, droplet digital PCR (ddPCR), microarrays, and RT-qPCR. RT-qPCR is widely used for targeted quantification because of its sensitivity and specificity, although assay design and normalization remain important sources of uncertainty [13].

Appropriate normalization can reduce technical variation associated with sample input and assay processing, but its effectiveness depends on the control and the processing stages it represents. Endogenous reference RNAs should be sufficiently stable across the relevant samples and biological groups [14]. The MIQE and MIQE 2.0 guidelines emphasize experimental justification of the choice and number of references; use of a single reference requires evidence of stability under the actual study conditions [15,16]. Reference stability can vary with tissue, disease, and experimental context [17]. An unstable normalization factor can exaggerate, attenuate, or obscure biological differences [18].

This review summarizes normalization strategies used for miRNA RT-qPCR in blood-derived samples in obesity research, compares their application across sample matrices, and outlines practical considerations for selecting reference RNAs and process controls.

The review focuses on the evidence supporting normalization practices in obesity-related blood-derived samples, taking account of sample matrix and cohort characteristics. Small matrix- or age-specific subgroups are treated as evidence gaps. Recommendations are qualified by the extent to which primary studies report candidate selection, stability testing, analytical performance, and validation in the relevant cohort. The frequencies describe the selected publications and do not establish analytical superiority or comprehensive coverage of the field.

### Literature Search and Study Selection

A literature search was conducted in PubMed (https://pubmed.ncbi.nlm.nih.gov/) and Wiley Online Library (https://onlinelibrary.wiley.com/), with the last searches performed in September 2026. The publications included in the quantitative survey span 2016–2026.PubMed searches combined free-text terms and Medical Subject Headings (MeSH), using the following query: ((“microRNAs”[MeSH Terms] OR “reference miRNAs” OR “housekeeping miRNAs”) AND (“obesity”[MeSH Terms] OR “obese”) AND (“RT-qPCR” OR “real-time PCR” OR “normalization” OR “reference genes”)) AND (“plasma” OR “blood” OR “serum” OR “leukocytes” OR “peripheral blood mononuclear cells” OR “PBMC” OR “whole blood”)).

The “Humans” filter was additionally applied in PubMed. In Wiley Online Library, the following search blocks were combined using AND: (miRNA* OR microRNA*) in Anywhere; (obesity OR obese) in Title; (“RT-qPCR” OR “real-time PCR”) in Anywhere; (blood OR plasma OR “peripheral blood mononuclear cells” OR PBMC) in Anywhere; (normalization OR normalized OR reference OR housekeeping) in Anywhere; and (Human OR men OR women OR patients OR children OR adolescents) in Anywhere.

Eligible studies reported RT-qPCR measurements of miRNAs in human blood-derived samples and provided information on normalization. Overweight or obesity had to define a study group or be explicitly investigated in relation to miRNA measurements; its presence solely as a descriptive cohort characteristic was insufficient. Studies involving comorbid conditions or mixed-weight cohorts were eligible when this requirement was met. For publications combining different sample types or experimental systems, only the relevant human blood-derived miRNA analyses were included.

Exclusion criteria comprised reviews, systematic reviews, meta-analyses, commentaries, conference abstracts, and editorials; studies restricted to animal models or cell lines; studies without RT-qPCR quantification of miRNAs; studies unrelated to overweight or obesity; and publications without extractable normalization information. Reports whose full texts could not be obtained through institutional access or open-access sources were recorded as unavailable for full-text assessment.

Studies were selected by assessment of their full texts against the eligibility criteria above. For each publication, the author and year, reference number, sample matrix, control RNAs, their reported roles, and evidence supporting reference selection and validation were recorded. Reported control use was distinguished from assessment of stability in the relevant cohort. Candidate controls, validation methods, and literature- or manufacturer-based selection rationales were recorded where available; unreported validation was distinguished from information that could not be verified. Methodological and biological background publications are cited separately and are not included in the frequency calculations. This narrative survey describes practices within the selected publication set and does not establish comparative superiority of normalization strategies.

## 2. Sources of Variability in Reference Small RNA RT-qPCR

Reference-RNA suitability depends on both molecular properties and the study context, including sample matrix, collection and storage conditions, and cohort characteristics. A control validated for one matrix cannot be assumed to be suitable for another. Whole blood contains erythrocytes, leukocytes, and platelets, each with a distinct miRNA profile; differences in cellular composition can therefore change the measured signal. A report by Abraham et al. identified hsa-let-7b and hsa-miR-423-3p as candidate references across the hematopoietic cell types examined [19]. These findings do not establish their stability in obesity-related comparisons. Experimental work has implicated let-7b-5p in metabolic regulation [20], and Toor et al. reported higher serum miR-423-3p levels in patients with acute ischemic stroke and type 2 diabetes than in stroke patients without diabetes [21]. Such findings illustrate why a reference selected in one setting requires validation before use in a different biological comparison.

Hemolysis can be assessed using the difference between the Cq values of miR-23a and erythrocyte-enriched miR-451: ΔCq = Cq(miR-23a) − Cq(miR-451). Blondal et al. described this approach as an indicator of erythrocyte-derived miRNA contamination and reported that ΔCq values >5 indicate possible contamination, whereas values of approximately 7-8 or higher indicate a high risk of hemolysis affecting the measured miRNA profile [22]. These thresholds were established for the assay conditions used in that study and should be interpreted in relation to the assay and sample processing protocol rather than applied as universal acceptance limits. Hemolysis assessment is particularly relevant when considering erythrocyte-associated miRNAs such as miR-16 as references.

Storage effects differ among candidate controls. Xiang et al. reported declining serum U6 levels after repeated freeze–thaw cycles, whereas miR-16 and miR-24 were comparatively stable under the conditions examined [23]. Aliquoting samples and limiting repeated freeze–thaw cycles can reduce avoidable handling variation; stability should nevertheless be assessed for the relevant matrix and storage protocol.

Obesity frequently coexists with metabolic disorders that may independently affect circulating miRNA levels. Reference validation should therefore consider obesity severity, comorbidities, and the intended group comparison. For example, lower circulating miR-216a levels were associated with metabolic alterations in women [24]. Participant characteristics, including age, can also influence blood miRNA profiles [25]. Appropriate cohort definition and consideration of age and sex are therefore important when evaluating candidate references.

Biological and processing-related variation can affect candidate reference RNAs differently across whole blood, blood-cell fractions, plasma, and serum. Reference selection should account for these sources of variation and be validated for the intended comparison.

## 3. Normalization Strategies

Accurate normalization of RT-qPCR data is a prerequisite for interpretable microRNA quantification. The normalization strategy should be selected according to the biological measurement being made and the technical steps that require control. Endogenous reference RNAs can provide a biological normalization factor when their stability is validated for the relevant matrix and cohort, whereas exogenous spike-in controls interrogate only the processing stages that occur after their addition. These functions are complementary but not interchangeable.

Endogenous controls used in miRNA RT-qPCR include small nuclear RNAs such as U6, small nucleolar RNAs such as RNU48, and miRNAs such as miR-16-5p. Their use requires evidence of stability in the relevant samples. For a single reference, ΔCq is the target Cq minus the reference Cq; interpretation of relative quantities must also account for amplification efficiency. No single reference is universally stable across study conditions [26].

Commercial miRNA assays may include recommended endogenous controls, but a manufacturer recommendation does not establish stability in a particular clinical cohort. Candidate references can also be selected using custom assays, including stem-loop and two-tailed reverse-transcription approaches [27,28]. These approaches offer flexibility in target selection; multiplex reverse transcription may reduce sample requirements, provided that the combined assay is validated.

For a validated multi-reference strategy, the normalization factor is calculated from the geometric mean of relative reference-RNA quantities [15,16,29,30]. Because Cq is logarithmically related to starting template quantity, geometrically averaging raw Cq values is not equivalent to geometrically averaging RNA quantities. For each assay, the same calibrator, such as a selected reference sample or pooled sample, is used across all samples as the baseline for relative quantity calculations. For assay *i*, with an amplification factor *E_i_* that is approximately constant across the relevant samples, the quantity of RNA *i* in sample *s* relative to this common calibrator is:

(1) $$Q_{i,s}={E_{i}}^{{Cq}_{i,cal}-{Cq}_{i,s}}$$

where *E_i_* is the fold amplification per cycle (2 for ideal doubling). The subscripts *cal* and *s* denote the calibrator and sample, respectively; *i* identifies the assay. A common calibrator is used across samples for each assay.

For *m* validated endogenous reference RNAs, the normalization factor is:

(2) $${NF}_{s}={\left(\prod_{j\;=\;1}^{m}Q_{j,s}\right)}^{\frac{1}{m}}$$

where $\overset{m}{\underset{j\;=\;1}{\prod }}Q_{j,s}$ is the product of the relative quantities of all *m* selected reference RNAs in sample *s*.

The normalized relative target quantity is:

(3) $$N_{t,s}=\frac{Q_{t,s}}{{NF}_{s}}$$

These are relative quantities rather than absolute copy numbers. Under a common amplification efficiency, geometric averaging of quantities corresponds to arithmetic averaging on the Cq scale with the appropriate calibrator adjustment; when efficiencies differ between assays, quantities should be calculated separately for each assay. The number of reference RNAs should be determined from validation results rather than fixed in advance. A single reference may be adequate only when its stability for the specific matrix, population, and biological contrast is convincingly demonstrated.

In parallel with endogenous references, exogenous synthetic microRNAs (spike-ins) are widely used. These are typically non-human RNAs, for example cel-miR-39 or ath-miR-159a. A spike-in monitors technical variation only in the processing steps that occur after the point at which it is added; it cannot retrospectively correct variation caused by blood collection, transport, storage, clotting, hemolysis, or cellular/platelet contamination that occurred beforehand. Accordingly, the point of addition and the intended function of each spike-in should be stated explicitly: quality assessment, identification of failed samples or reactions, monitoring or correction of RNA recovery, or direct use in a normalization calculation. Consistent spike-in recovery does not establish that the endogenous RNA pool was comparable before spike-in addition, and combined use of exogenous and endogenous controls does not imply that both should automatically be combined in a single normalization denominator. Endogenous reference RNAs remain necessary when the analytical objective requires normalization to a stable biological reference, provided that their suitability has been validated for the specific study context.

Endogenous references require validation of biological stability, whereas exogenous spike-ins require assessment of technical performance and recovery for their intended role. The following section describes the methods used to evaluate candidate endogenous references.

## 4. Evaluation of Candidate Normalizers

Several algorithms are used to evaluate candidate reference RNAs (Table 1), including geNorm [30], NormFinder [31], BestKeeper [32], the comparative ΔCt method [33], and RefFinder [34]. geNorm evaluates pairwise expression ratio stability and the effect of adding further reference genes; NormFinder uses a model-based variance approach that can incorporate group structure; BestKeeper evaluates candidate consistency using Cq-based statistics and correlations with a BestKeeper index; the BestKeeper index is an intermediate statistic for candidate evaluation and does not justify geometric averaging of raw Cq values for general normalization; the comparative ΔCt method evaluates pairwise ΔCq variability; and RefFinder aggregates rankings from several component methods. These tools provide relative assessments within the candidate panel and do not by themselves prove that the highest-ranked candidate is biologically invariant between obesity and control groups. Candidates that co-vary with phenotype can retain stable pairwise ratios, and correlation with the BestKeeper index does not establish between-group equivalence. RefFinder will still return a top-ranked candidate even when all supplied candidates are unsuitable. Therefore, validation should evaluate candidates jointly across all relevant groups, explicitly consider group structure, inspect the magnitude and uncertainty of between-group shifts, consider potential co-regulation, and allow rejection of the entire candidate panel. Disagreement among algorithms should prompt examination of group shifts, outliers, assay performance, and model assumptions rather than automatic rank aggregation. Method-specific thresholds are useful guides, not universal evidence of biological stability.

These methods support reference evaluation when their inputs and assumptions match the experimental design.

## 5. Current Practices in Obesity Research

The 32 publications in Table 2 yielded 33 matrix analysis records. Of these 33 records, 30 records from 29 publications had an assigned matrix: six PBMC, one whole blood, ten plasma, two plasma-derived extracellular vesicle, and eleven serum records. The remaining three publications had unresolved matrix assignments and were retained in Table 2 but excluded from matrix-specific frequencies. Reported control use is distinct from reference-RNA validation. These publications document reported normalization practice; they were not all designed to select or validate endogenous references.

**Table 2:** Reported normalization and control practices in 32 publications related to overweight and obesity, comprising 33 matrix analysis records.

Author, Year	Age Group	Blood-Derived Sample Matrix	Endogenous Reference(s)	Exogenous/Synthetic Controls *	Evidence for Control Selection, Role, and Validation
Mazloom et al., 2016 [35]	Adults	PBMCs	U6	-	U6 use in PBMCs is confirmed by the figure legend. The selection rationale and any within-study stability assessment could not be verified from the accessible text.
Pergoli et al., 2017 [36]	Adults	Plasma-derived extracellular vesicles	U6	ath-miR159a (N)	geNorm and NormFinder supported global mean screening normalization. Targeted validation combined RNU6 and ath-miR159a. RNU48 and four miRNAs were also evaluated as alternative strategies; RNU44 was a panel control. Alternative strategies and panel membership are not counted as final selected reference use.
Ghorbani et al., 2018 [37]	Adults	Serum	miR-16	cel-miR-39 (Q)	miR-16 was the serum reference; cel-miR-39 monitored extraction. Comparable spike-in measurements support technical consistency. A study-specific stability assessment of miR-16 is not described.
Yu et al., 2018 [38]	Adults	Serum	U6	-	U6 is the reported miRNA reference, and the serum assay refers to the same RT-qPCR procedure. Serum-specific reference selection and stability evidence could not be verified.
Wang et al., 2018 [39]	Adults	Blood fraction unresolved	U6	-	No U6 stability assessment is described. The blood fraction used for the clinical RNA assay is unclear; this publication is excluded from matrix-specific counts.
Marzano et al., 2018 [40]	9-15 years	Serum	miR-191	-	geNorm and NormFinder assessed miR-26b, miR-182, miR-191 and miR-423; miR-191 was used for RT-qPCR normalization. Serum is specified in the assay-specific validation section, which takes precedence over the broader blood collection description.
Russo et al., 2018 [41]	Adults	Plasma	-	cel-miR-39 (N)	Plasma expression was calculated as 2^−ΔCt^, with ΔCt = Ct(target) − Ct(cel-miR-39). No endogenous reference was selected for this plasma analysis.
Miao et al., 2019 [42]	Adults	Plasma	-	cel-miR-39 (N)	cel-miR-39 was added during plasma RNA extraction and explicitly used as the denominator in the relative expression calculation. No endogenous reference selection or stability assessment is described.
Manning et al., 2019 [43]	Adults	Plasma	-	cel-miR-39-3p (N)	cel-miR-39-3p was introduced during plasma RNA extraction and used for RT-qPCR normalization, following earlier protocols. Quality controls for the separate NanoString analysis do not constitute endogenous RT-qPCR reference validation.
Rubie et al., 2019 [44]	Adults	PBMCs	RNU48	-	RNU48 (assay 001006) was explicitly assigned to miR-496 normalization in PBMCs. No study-specific stability assessment is described.
Tryggestad et al., 2019 [45]	Adults	PBMCs	U6	-	U6 was used for cellular miRNA normalization in PBMCs. Summary Cq values are reported, but no between-group assessment of U6 stability is described.
Tryggestad et al., 2019 [45]	Adults	Serum		cel-miR-39 (N)	cel-miR-39 was used for serum miRNA normalization. The methods distinguish this serum control from U6, which was used for cellular miRNAs.
Sun et al., 2019 [46]	Adults	Plasma	Sno202	-	Sno202 is named in a shared protocol for mouse kidney and human plasma. Its use in the human plasma assay is not unambiguously established. It is retained as an unresolved assignment and excluded from confirmed control-use counts.
Fu et al., 2019 [47]	Adults	Serum	-	cel-miR-39 (N)	The methods explicitly distinguish cel-miR-39 for serum miRNAs from U6 for tissue and cellular miRNAs. No endogenous serum reference is selected; tissue and cellular controls are not transferred to the serum analysis.
Cabiati et al., 2020 [48]	12-13 years	Plasma	-	cel-miR-39 (N; also technical control)	cel-miR-39 served as a technical control and was used in ΔΔCt normalization of plasma miRNAs. It is counted once under normalization. No endogenous reference selection or stability assessment is described.
Cheng et al., 2020 [49]	Adults	Serum	U6	-	U6 is explicitly identified as the serum miR-214 reference, with 2^−ΔΔCt^ quantification. No comparison of candidate references or assessment of U6 stability in the study samples is described.
Sørensen et al., 2021 [50]	Adults	Serum	U6	ath-miR-159 (N); cel-miR-39 (N)	U6 was combined with ath-miR-159 and cel-miR-39, added at extraction and reverse transcription, respectively. No U6 stability assessment is described. The source does not clearly specify the scale of geometric averaging; its calculation is not endorsed here.
Bellae Papannarao et al., 2022 [51]	Adults	Plasma	miR-16, miR-24	-	miR-16 and miR-24 were used as endogenous controls and reported to have comparable expression between lean and obese groups. This provides limited within-study consistency evidence; formal ranking against alternative candidates is not described.
Abdallah et al., 2022 [52]	Adults	Plasma	SNORD68, RNU6B (U6)	-	The supplementary methods state that the average of SNORD68 and RNU6B was used as the endogenous control. No candidate comparison or stability assessment is described, and the averaging scale is not explicitly specified.
Gholami et al., 2022 [53]	Adults	PBMCs	SNORD44; SNORD47 (separate analyses)	-	Results are reported separately with SNORD44 and SNORD47. This does not establish their combination in one normalization factor. No study-specific reference stability ranking or selection procedure is described.
Ojeda-Rodríguez et al., 2022 [54]	7-16 years	Plasma	miR-425-5p	cel-miR-39 (Q); UniSp6 (Q)	NormFinder assessed intra- and intergroup variation and selected miR-425-5p for targeted analysis. Screening used global mean normalization. cel-miR-39 and UniSp6 were technical controls, not the selected endogenous normalization reference.
Dai et al., 2022 [55]	Adults	Serum	U6	-	No stability assessment is described. The presumed U6–miRNA assignment is excluded from confirmed control-use counts.
Xu et al., 2022 [56]	Adults	Serum	-	cel-miR-39-3p (N)	cel-miR-39-3p is explicitly identified as the reference for serum miR-149-3p. The spike-in addition stage is not clearly specified. No endogenous reference selection or stability validation is described.
Michalak-Stoma et al., 2023 [57]	Adults	PBMCs	RNU48	-	RNU48 is identified as the PBMC miRNA reference. No study-specific candidate comparison or stability assessment is described. Technical replication does not itself establish biological reference stability.
Gil-Martínez et al., 2023 [58]	Adults	Serum	miR-103a-3p, miR-191-5p, U6	cel-miR-39-3p (Q); UniSp6 (Q)	Between-group Cq comparisons and BestKeeper were used to evaluate controls, but the final endogenous reference set for serum is not explicitly resolved. Candidate endogenous controls are excluded from confirmed normalization counts; the stated reverse-transcription controls are counted as Q.
Behrooz et al., 2023 [59]	10-18 years	PBMCs	RNU24	-	RNU24 was selected based on previous reports of PBMC stability. A new within-study reference comparison is not described. Efficiency-corrected relative quantification specifies the calculation, rather than providing new evidence of reference stability.
Santos et al., 2023 [60]	5-9 years	Plasma	miR-16-5p	cel-miR-39-3p; ath-miR-159 (tested and rejected)	miR-16-5p was used because Cq values were reported as stable between groups. cel-miR-39-3p and ath-miR-159 were tested but excluded for unreliable amplification; they are not counted as retained normalization or QC controls.
Dadkhah Nikroo et al., 2024 [61]	Adults	Serum/plasma unresolved	miR-16	cel-miR-39-1 (Q; matrix unresolved)	miR-16 normalized circulating miR-141; cel-miR-39-1 monitored RNA recovery, not cDNA synthesis alone. Serum/plasma terminology is inconsistent. Both occurrences are retained but excluded from matrix-specific counts.
Li et al., 2025 [62]	Adults	Plasma-derived extracellular vesicles	U6	-	U6 normalized selected plasma exosomal miRNAs in RT-qPCR. No U6 stability assessment is described. Confirmation of sequencing-derived target miRNAs is not validation of the normalization reference.
Masoumi-Ardakani et al., 2025 [63]	Adults	Serum	-	cel-miR-39 (N; also recovery control)	cel-miR-39 was used with the extraction kit procedure to monitor recovery and as the reference for serum miR-33b. It is counted once under normalization. No endogenous reference selection or stability assessment is described.
Li et al., 2025 [64]	Adults	Plasma	miR-16-5p	-	The figure legend identifies miR-16 as the internal reference. No endogenous reference selection or stability assessment is described.
Padro et al., 2026 [65]	Adults	Whole blood	miR-16-5p	cel-miR-39 (N)	Both miR-16-5p and cel-miR-39 are explicitly described as normalization controls. Detection and amplification quality criteria are reported, but biological reference stability between conditions is not established. cel-miR-39 is therefore not classified as QC-only.
Topal et al., 2026 [66]	9-15 years	Plasma/cellular fraction unresolved	U6	-	No U6 stability assessment is described. Plasma and cellular fractions are collected, but the fraction used for RNA extraction is not identified; matrix-specific counts exclude this publication.

**Figure 1:**
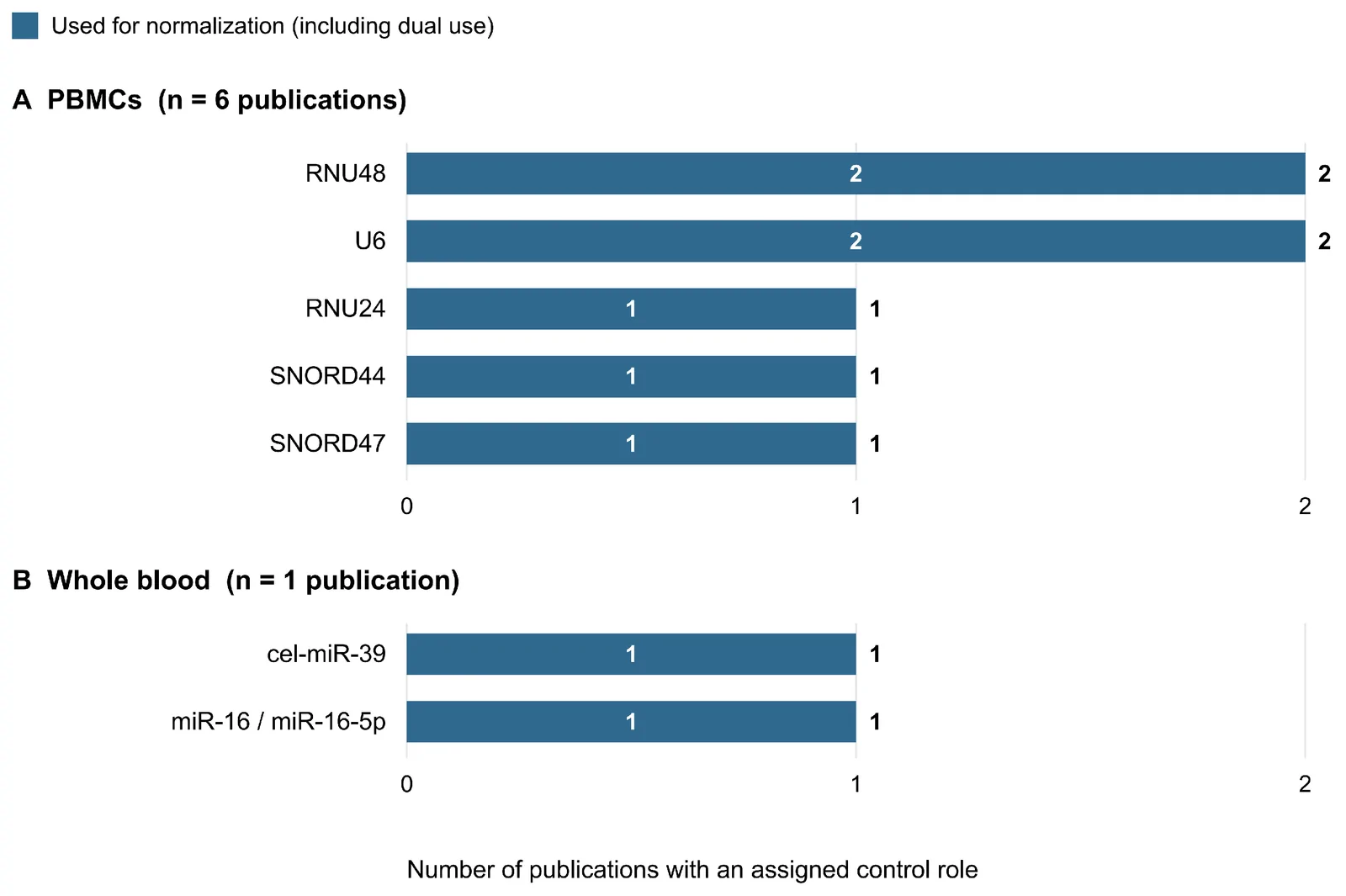
Confirmed control use in PBMCs ((**A**); six publications) and whole blood ((**B**); one publication). Bars count distinct publication–matrix–control combinations. Controls in separate normalization analyses can each contribute one occurrence. This figure describes reported control use, not validation or analytical suitability. Matrix-unresolved publications are excluded; reported assignments and their qualifications are summarized in Table 2.

* N, used in the reported normalization calculation, including controls with an additional technical monitoring role; Q, technical quality control only. Each retained control contributes one occurrence per publication and sample matrix, irrespective of repeated assay stages. A control used for both normalization and technical monitoring is counted once under N; QC-only use is counted under Q. Unresolved assignments, rejected controls, and alternative screening strategies are retained explicitly where relevant but excluded from confirmed control-use counts. For descriptive aggregation, U6/RNU6/RNU6B, miR-16/miR-16-5p, and the reported cel-miR-39 and ath-miR-159 variants are grouped under the figure labels. This grouping does not establish sequence, assay, or biological equivalence. References [35,36,37,38,39,40,41,42,43,44,45,46,47,48,49,50,51,52,53,54,55,56,57,58,59,60,61,62,63,64,65,66] comprise the 32-publication survey. Figure 1 summarizes assigned control use in PBMCs and whole blood separately. Among six PBMC publications, U6 and RNU48 each occur in two; SNORD44, SNORD47 and RNU24 each occur in one. There are 32 publications and 33 matrix analysis records because Tryggestad et al. [45] contributed separate PBMC and serum analyses.The whole-blood study by Padró et al. [65] reports both miR-16-5p and cel-miR-39 as normalization controls. Wang et al. [39] and Topal et al. [66] are excluded from the matrix-specific cellular and plasma categories, respectively, because the blood fraction used for RNA extraction is insufficiently specified.

**Figure 2:**
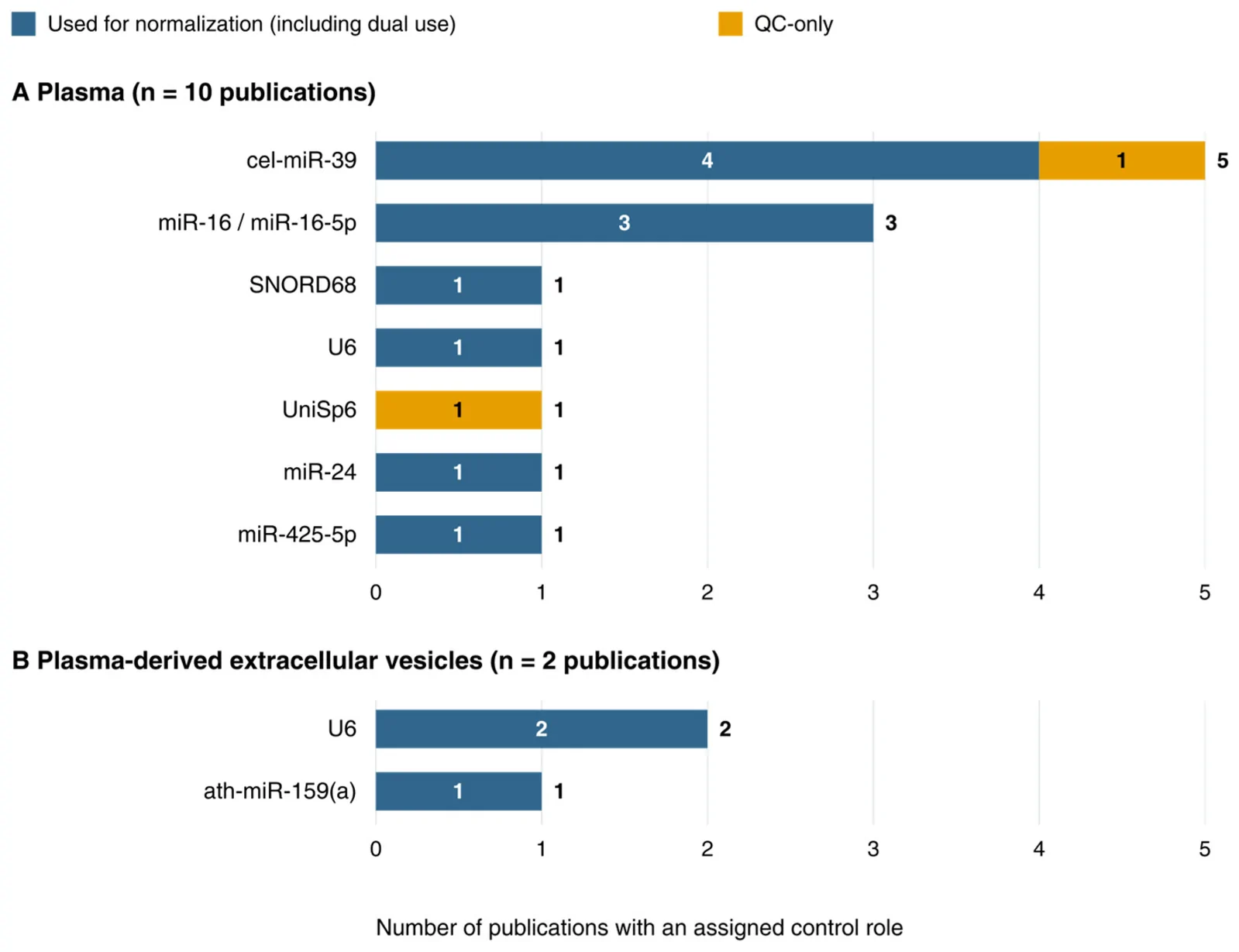
Confirmed control use in plasma ((**A**); ten publications) and plasma-derived extracellular vesicle samples ((**B**); two publications). Across both panels, cel-miR-39 occurs in five publications (four normalization uses, N, and one QC-only use, Q); U6 and miR-16/miR-16-5p each occur in three. Bars represent absolute counts of distinct publication–matrix–control combinations. Panel denominators include publications with an assigned matrix even when the final control assignment is unresolved. Alternative or rejected controls and matrix- or role-unresolved occurrences are excluded. The contributing publications and relevant qualifications are summarized in Table 2.

Plasma and plasma-derived extracellular vesicle samples are summarized separately in Figure 2. Across the ten plasma and two vesicle publications, cel-miR-39 has five retained occurrences: four normalization uses (Russo et al. [41], Miao et al. [42], Manning et al. [43] and Cabiati et al. [48]) and one QC-only use (Ojeda-Rodríguez et al. [54]). U6 occurs in three normalization reports (Pergoli et al. [36], Abdallah et al. [52] and Li Y et al. [62]), and the combined miR-16/miR-16-5p category also occurs in three (Bellae Papannarao et al. [51], Santos et al. [60] and Li J et al. [64]). Dadkhah Nikroo et al. [61] is excluded from this matrix-specific tally because of inconsistent serum/plasma terminology; the corresponding miR-16 and cel-miR-39 assignments remain explicitly qualified in Table 2. Sno202 in Sun [46] is excluded because its role in the human plasma assay is unclear. The rejected spike-ins in Santos [60] are not counted as retained controls.

**Figure 3:**
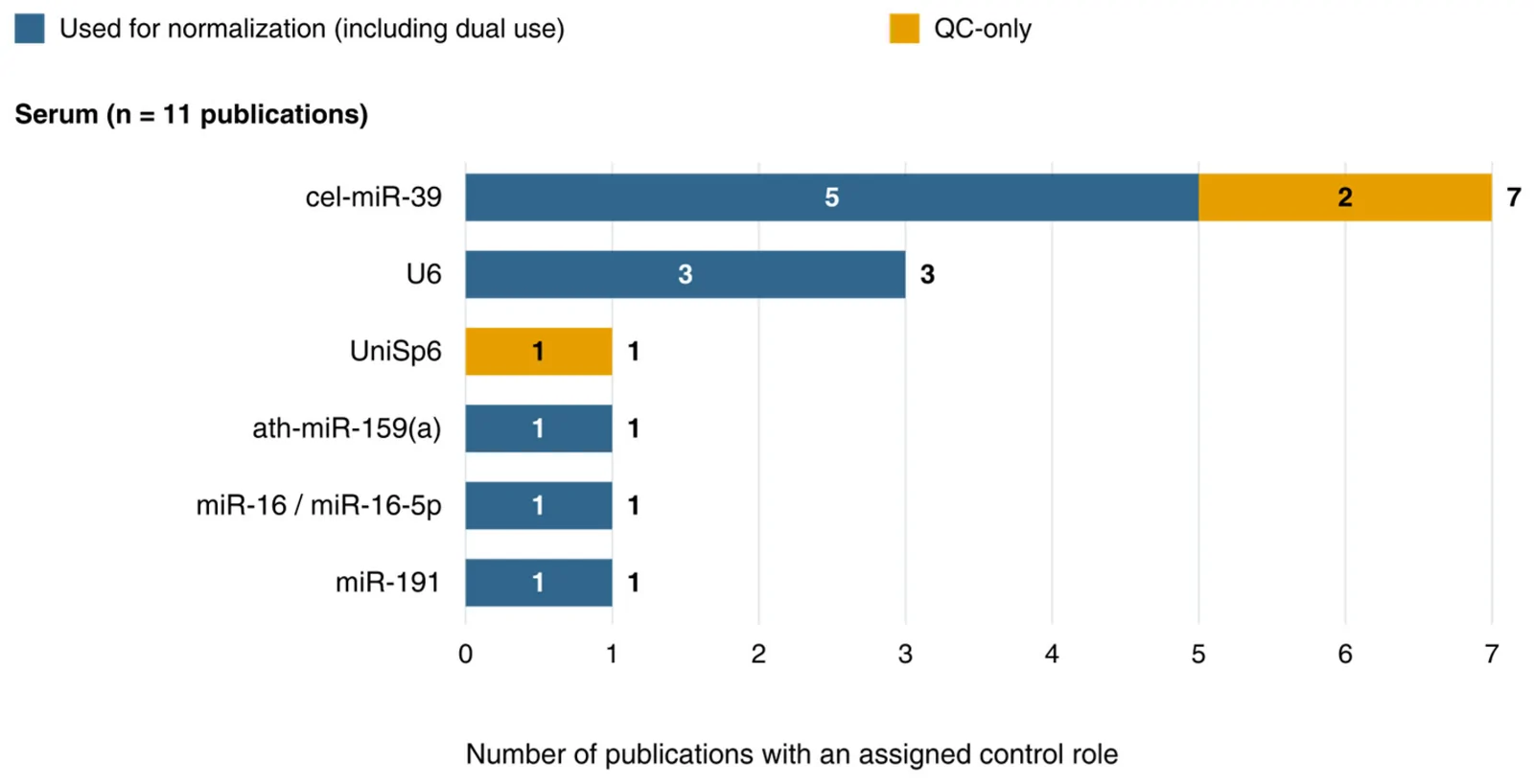
Confirmed control use in serum (eleven publications). cel-miR-39 occurs in seven publications: five normalization uses (N; Tryggestad et al. [45], Fu et al. [47], Sørensen et al. [50], Xu [56] and Masoumi-Ardakani et al. [63]) and two QC-only uses (Q; Ghorbani et al. [37] and Gil-Martínez et al. [58]). U6 has three confirmed normalization uses (Yu et al. [38], Cheng et al. [49] and Sørensen et al. [50]). The unresolved U6 assignment in Dai et al. [55] and the incompletely specified final endogenous reference set in Gil-Martínez et al. [58] are excluded from normalization counts. miR-191 is retained under its reported name in Marzano et al. [40]. Bars represent absolute counts of distinct publication–matrix–control combinations. N denotes reported normalization use, including controls with an additional technical monitoring role; Q denotes QC-only use. The denominator includes serum publications with an assigned matrix, including those with unresolved final control assignments.

Serum requires separate consideration because clot formation can alter its miRNA composition. Obtaining serum involves clot formation, and residual leukocytes and platelets can release microRNAs during clotting and clot retraction, potentially altering the measured endogenous RNA profile [67]. Strict standardization of collection, clotting time, temperature, and centrifugation is therefore required. A cel-miR-39 spike-in added only after clotting and centrifugation cannot assess or correct endogenous miRNA release, loss, degradation, hemolysis, or cellular/platelet contamination that occurred before its addition; it can monitor only the downstream stages to which it is actually exposed. Reference use in serum samples is summarized in Figure 3.

The age-stratified counts in Table 3 are generated from the same included occurrence records. Among nine adult plasma/plasma-derived-vesicle publications, U6 occurs in three and miR-16/miR-16-5p in two. The latter excludes the matrix-unresolved Dadkhah Nikroo et al. report [61]; the third confirmed occurrence in the overall plasma-related category is the pediatric Santos et al. study [60]. Pediatric studies are summarized as one broad group rather than overlapping age bands. These small, heterogeneous subgroups describe reported practice and do not support age-specific rankings of analytical suitability.

Excluded from matrix-specific counts are Wang et al. [39], Dadkhah Nikroo et al. [61], and Topal et al. [66]. Unresolved control assignments in Sun et al. [46], Dai et al. [55], and Gil-Martínez et al. [58] are also excluded. These exclusions reflect uncertainty in the reported methods rather than evidence that a control was absent or unsuitable.

The cohorts encompass type 2 diabetes, insulin resistance and other conditions associated with obesity, including dyslipidemia, hypercholesterolemia, coronary artery disease, asthma, colorectal cancer, fatty liver disease, nephropathy, polycystic ovary syndrome and gestational diabetes. The heterogeneous cohort definitions and control validation practices preclude disease-specific recommendations based on control-use counts alone.

Padró et al. [65] was retained because its exploratory miRNA analysis involved adults with overweight or class I obesity; the control assignments refer to its whole-blood assay. Controls used in unrelated tissues, cell experiments, or mRNA assays are not assigned to the human blood-derived miRNA analysis.

U6 and miR-16/miR-16-5p recur across several sample matrices, but their use does not establish suitability in every context. Members of the miR-15/16 family participate in apoptosis, proliferation, and differentiation, illustrating that these RNAs have regulated biological functions [68,69,70]. Lee et al. reported reduced miR-16 expression in skeletal muscle models of lipid-induced insulin resistance [71]. This finding does not directly establish instability in blood, but it supports the need to evaluate possible phenotype-associated regulation in the intended matrix. U6 is a spliceosomal RNA with a different cellular role and biogenesis from miRNAs [72]. Its variable serum abundance and sensitivity to repeated freeze-thaw cycles have been documented [23]; these observations should not be generalized to all sample types. Reference suitability therefore requires assessment in the relevant matrix, cohort, and biological comparison.

Reference-RNA validation is heterogeneous across the retained publications. Four reports describe algorithm-based assessment of endogenous candidates or normalization strategies: Pergoli et al. [36], Marzano et al. [40], Ojeda-Rodríguez et al. [54], and Gil-Martínez et al. [58]. Pergoli et al. compared normalization strategies that included endogenous candidates and global mean normalization; this is distinguished from selection of a single endogenous reference. Two additional reports, Bellae Papannarao et al. [51] and Santos et al. [60], describe between-group consistency of the selected references, which provides more limited evidence than a formal candidate stability analysis. These assessments do not establish universal reference suitability, and the final serum reference set in Gil-Martínez et al. [58] remains incompletely specified.

Three publications explicitly report using more than one endogenous reference: Bellae Papannarao et al. [51], Abdallah et al. [52], and Gholami et al. [53]. Gholami et al. presents separate reference-normalized analyses, so this count does not imply that all three studies used a combined normalization factor. Abdallah et al. explicitly reports averaging SNORD68 and RNU6B, whereas the calculation scale is not specified. The unresolved final endogenous set in Gil-Martínez et al. [58] is not counted as confirmed use of a specified multi-reference set.

One publication, Behrooz et al. [59], explicitly justifies endogenous reference selection by previous PBMC stability studies, without describing a new within-cohort candidate comparison. No report was assigned to an explicitly manufacturer-selected endogenous reference category on the basis of the reviewed descriptions. Manufacturer-guided exogenous spike-in protocols, including Masoumi-Ardakani et al. [63], are classified separately. Unspecified selection rationales are not assumed to reflect prior validation or manufacturer recommendations. The validation status verification limits for Mazloom et al. [35], Yu et al. [38], and Li J et al. [64] remain explicit in Table 2. These overlapping reporting categories should not be interpreted as mutually exclusive groups or as proof that an unreported assessment was not performed.

Exogenous control roles are identified for the relevant assays in Table 2. The mutually exclusive plotting categories distinguish reported normalization use, including dual use, from QC-only use. Thus, a spike-in used both to monitor recovery and in the final calculation contributes only once to the normalization category. Rejected spike-ins and unresolved roles are retained for traceability but omitted from confirmed-use bars.

The available evidence is insufficient to rank normalization strategies by analytical performance. Candidate panels, stability metrics, processing protocols, and biological contrasts differ across studies, and several reports provide little information on reference selection. Comparable estimates of normalization bias and reproducibility are therefore lacking for many strategies. The practical recommendations below focus on defining the measurement basis, controlling relevant processing steps, evaluating phenotype-associated shifts, and reporting evidence for the final normalization factor.

## 6. Practical Workflow

The following workflow provides a framework for validation. Candidate selection, sample size, and the final number of reference RNAs should be justified for the study objective and observed performance:

1. Define the intended biological measurement and its denominator before selecting a normalization strategy (for example, miRNA abundance per unit starting biofluid volume, per specified cell number, or relative to a validated endogenous reference factor).
2. Define the matrix-specific input and processing chain separately: starting sample volume, cell number/composition where relevant, extracted RNA mass, eluate volume, and the fractions entering reverse transcription and PCR are not interchangeable quantities.
3. For each exogenous spike-in, specify the addition stage and intended role: quality assessment, identification of failed samples or reactions, recovery correction, or inclusion in the final normalization calculation. Define any sample exclusion rules in advance. Recovery correction requires evidence that the spike-in adequately represents the relevant technical variation.
4. Assemble candidate reference RNAs from relevant literature and/or discovery data while screening for known or plausible association with the phenotype under study and for potential co-regulation among candidates.
5. Evaluate analytical performance and expression stability across all relevant study groups using complementary approaches, such as geNorm, NormFinder, BestKeeper, and comparative ΔCt, while explicitly considering group structure and assay-specific amplification efficiency.
6. Assess the magnitude and uncertainty of between-group shifts in each candidate and in the proposed normalization factor. Define acceptable normalization bias for the intended biological comparison before evaluating the results. A nonsignificant group difference does not establish stability. Where equivalence testing is used, justify the equivalence margins in advance; imprecise estimates should be regarded as inconclusive.
7. Resolve discordant algorithmic rankings by examining group effects, outliers, technical performance, and method assumptions. The workflow must allow rejection of the entire candidate panel when no candidate is sufficiently supported.
8. Accept the final normalization factor only when analytical performance and within- and between-group stability satisfy the predefined requirements. Evaluate whether normalization could remove or introduce the phenotype-associated difference being measured. Determine the number of retained references from these results; if no candidate panel is adequately supported, expand the panel or revise the measurement strategy.

For plasma and serum, equalizing total RNA mass does not automatically preserve an interpretation as abundance per unit starting biofluid volume because differences in recovered RNA may have a biological component. For whole blood, cell composition is part of the measurement context; for PBMCs, cell number and cellular composition should be considered. For extracellular-vesicle-enriched preparations, the starting material, isolation procedure, and chosen normalization basis should be defined explicitly, and measurements per starting volume and per particle should not be treated as interchangeable.

Validation sample size should be justified by the study objective, expected variability, acceptable normalization bias, required precision, and design structure. No universal minimum number of samples or candidate references is prescribed. The aim is to obtain sufficiently precise estimates of analytical performance and within- and between-group stability for the intended biological comparison.

Input standardization should identify the quantity held constant. Equal starting plasma volume, RNA mass, eluate volume, and cDNA input control different sources of variation and may change the biological interpretation. Standardization at one stage does not eliminate all input-related contributions to Cq differences. Reagent lots, extraction procedures, reverse-transcription conditions, and PCR conditions should be kept as consistent as practicable within the validation experiment.

The final report should provide the raw or minimally processed stability metrics relevant to the methods used, amplification efficiency information, the rationale for the selected normalization factor, and information on key sample quality variables such as hemolysis and storage. Method-specific thresholds should be presented as guides rather than universal acceptance criteria. The analysis should also state whether any candidate panel was rejected and how uncertainty in the final normalization choice was handled.

## 7. Conclusions and Future Perspectives

This narrative review describes normalization practices reported in 32 publications dated 2016-2026 that investigated miRNA expression in blood-derived samples in the context of overweight, obesity, and obesity-associated conditions. It identifies substantial heterogeneity across biological matrices, cohorts, and control strategies. The collection includes studies that used reference RNAs for obesity-related or obesity-associated miRNA measurements, as well as a smaller group that evaluated reference candidates or normalization strategies. Reported control use and direct assessment of reference suitability represent different levels of evidence. U6 and miR-16/miR-16-5p recur in the retained literature, but frequency of use does not establish stability or analytical superiority. Interpretation is limited to the defined obesity-related publication set and the available evidence supporting each control assignment.

No analyzed reference can be considered a universal endogenous control for obesity-related miRNA studies across all blood-derived materials and study conditions. Candidate validation should combine analytical assessment with explicit evaluation of the intended biological contrast. geNorm, NormFinder, BestKeeper, comparative ΔCt, or RefFinder can support this process, but agreement among rankings does not exclude phenotype-associated co-regulation, and a top-ranked candidate is not necessarily suitable. The final normalization factor and the number of references should therefore be determined empirically, with the option to reject the entire candidate panel when the evidence is insufficient.

The proposed workflow is intended as a planning framework rather than a set of universal numerical thresholds for obesity-related miRNA studies. It emphasizes definition of the measurement basis, matrix-specific input and quality controls, transparent separation of exogenous process controls from endogenous normalization references, and reporting of validation evidence and uncertainty. These principles are intended to improve reproducibility without overstating what can be inferred from the limited and heterogeneous obesity-related literature.

Future research in obesity-related miRNA studies should evaluate candidate reference panels for specific blood-derived matrices and cohorts, compare normalization strategies in the same samples, and assess reproducibility across laboratories. Larger pediatric cohorts and better-defined normalization procedures for extracellular vesicle preparations would address important evidence gaps. Sharing raw Cq values, amplification efficiency estimates, and detailed processing protocols would facilitate independent evaluation. Reports should distinguish controls used for quality assessment, recovery correction, and final normalization and state whether endogenous reference stability was directly assessed.

## Figures and Tables

**Table 1:** Interpretation and limitations of common reference-RNA stability methods.

Method	Key Metrics *	Interpretation
geNorm [30]	M value: lower values indicate greater stability. V_n_/_n+1_ evaluates whether adding another reference improves the normalization factor; V < 0.15 is commonly used as an approximate guide.	«M» summarizes pairwise variation in expression ratios. «V» evaluates the effect of adding an additional reference. Neither metric excludes co-regulation or establishes stability between biological groups. The V = 0.15 threshold should be treated as guidance rather than a universal acceptance criterion.
NormFinder [31]	Stability value: lower values indicate greater stability; no universal cutoff.	The model estimates expression variation and can incorporate group structure, allowing assessment of within- and between-group components. A lower stability value indicates better relative performance within the supplied candidate panel and study design.
BestKeeper [32]	SD of Cq values: lower values indicate greater consistency; SD < 1.0 is commonly used as a screening criterion. Pearson correlation (r) with the BestKeeper index: higher positive correlation indicates greater concordance; the associated *p*-value describes statistical significance.	Cq dispersion and correlation with the BestKeeper index describe candidate consistency. A low SD and strong correlation indicate relative stability within the tested panel, but a significant correlation does not establish equivalent expression between biological groups.
Comparative ΔCt [33]	Mean SD of pairwise ΔCq values: lower values indicate greater stability; no universal cutoff.	Pairwise ΔCq variability assesses relative concordance among candidate references. Shared phenotype-associated shifts may remain undetected if candidate RNAs change in parallel.
RefFinder [34]	Comprehensive ranking based on the geometric mean of rank weights from the component methods; a lower comprehensive ranking value/rank 1 indicates the highest relative stability. No universal cutoff.	RefFinder integrates rankings from component methods such as geNorm, NormFinder, BestKeeper, and comparative ΔCt. The highest-ranked candidate may nevertheless be unsuitable if the entire candidate panel lacks adequate biological stability.

* The metrics in Table 1 describe relative performance within the tested panel. No score, correlation, threshold, or rank alone establishes that a reference is sufficiently stable for the intended biological comparison.

**Table 3:** Age-stratified confirmed control use derived from the qualified assignments in Table 2. Values are numbers of publications; bracketed numbers identify the contributing references. N includes normalization with or without an additional technical control role; Q denotes QC-only use.

Blood-Derived Sample Category	Age Group	Publications, n	Endogenous Reference(s)	Exogenous/Synthetic Controls
Whole blood/PBMCs	Adults	6	RNU48: 2 [44,57] U6: 2 [35,45] SNORD44: 1 [53] SNORD47: 1 [53] miR-16/miR-16-5p: 1 [65]	cel-miR-39: 1 [65] (N)
Whole blood/PBMCs	Children/adolescents	1	RNU24: 1 [59]	None assigned
Plasma/plasma EVs	Adults	9	U6: 3 [36,52,62] miR-16/miR-16-5p: 2 [51,64] SNORD68: 1 [52] miR-24: 1 [51]	cel-miR-39: 3 [41,42,43] (N) ath-miR-159(a): 1 [36] (N)
Plasma/plasma EVs	Children/adolescents	3	miR-16/miR-16-5p: 1 [60] miR-425-5p: 1 [54]	cel-miR-39: 1 [48] (N) cel-miR-39: 1 [54] (Q) UniSp6: 1 [54] (Q)
Serum	Adults	10	U6: 3 [38,49,50] miR-16/miR-16-5p: 1 [37]	cel-miR-39: 5 [45,47,50,56,63] (N) cel-miR-39: 2 [37,58] (Q) UniSp6: 1 [58] (Q) ath-miR-159(a): 1 [50] (N)
Serum	Children/adolescents	1	miR-191: 1 [40]	None assigned

## Data Availability

No new data were created or analyzed in this study. Data sharing is not applicable to this article.

## Abbreviations

The following abbreviations are used in this manuscript:

RT-qPCR	Reverse transcription quantitative PCR
MIQE	Minimum Information for Publication of Quantitative Real-Time PCR Experiments
ddPCR	Droplet digital PCR
SD	Standard deviation
Cq	Quantification cycle
MeSH	Medical Subject Headings
PBMCs	Peripheral blood mononuclear cells
T2DM	Type 2 diabetes mellitus
EVs	Extracellular vesicles
QC	Quality control
snRNA	Small nuclear RNA
NF	Normalization factor
